# Non-hematopoietic Control of Peripheral Tissue T Cell Responses: Implications for Solid Tumors

**DOI:** 10.3389/fimmu.2018.02662

**Published:** 2018-11-15

**Authors:** Ryan S. Lane, Amanda W. Lund

**Affiliations:** ^1^Department of Cell, Developmental and Cancer Biology, Oregon Health and Science University, Portland, OR, United States; ^2^Department of Molecular Microbiology and Immunology, Oregon Health and Science University, Portland, OR, United States; ^3^Department of Dermatology, Oregon Health and Science University, Portland, OR, United States; ^4^Knight Cancer Institute, Oregon Health and Science University, Portland, OR, United States

**Keywords:** non-hematopoietic cells, T cell, immunotherapy, extravasation, interstitial migration, fluid flow, trafficking

## Abstract

In response to pathological challenge, the host generates rapid, protective adaptive immune responses while simultaneously maintaining tolerance to self and limiting immune pathology. Peripheral tissues (e.g., skin, gut, lung) are simultaneously the first site of pathogen-encounter and also the location of effector function, and mounting evidence indicates that tissues act as scaffolds to facilitate initiation, maintenance, and resolution of local responses. Just as both effector and memory T cells must adapt to their new interstitial environment upon infiltration, tissues are also remodeled in the context of acute inflammation and disease. In this review, we present the biochemical and biophysical mechanisms by which non-hematopoietic stromal cells and extracellular matrix molecules collaborate to regulate T cell behavior in peripheral tissue. Finally, we discuss how tissue remodeling in the context of tumor microenvironments impairs T cell accumulation and function contributing to immune escape and tumor progression.

## Introduction

Immune surveillance and protective immunity is dependent upon sequential, rapid activation, and mobilization of hematopoietic cells that undergo multiple intercellular interactions to mediate immune control. Rather than being stochastic, these interactions are guided by non-hematopoietic cells that generate and maintain tissue scaffolds. The microenvironments through which leukocytes traffic (e.g., blood, lymphoid organs, peripheral tissues) differ significantly with respect to cellular and protein composition and remodel in the context of disease and with age. Thus, in order to perform their protective function, both effector and memory T cells must adapt to continuously changing physical, biochemical, and metabolic tissue environments.

*De novo* T cell priming is initiated in lymph nodes (LN) that drain peripheral sites of infection, inflammation, and tumors. Within lymphoid organs, non-hematopoietic cells direct cellular interactions and increase the probability of immune activation. Lymph-borne antigen is transported to LNs through afferent lymphatic vessels that connect to the subcapsular sinus allowing delivery of large particulate antigens (>70 kDa) to interfollicular dendritic cells (DC) and subcapsular macrophages (1, 2). Small antigens (<70 kDa) enter fibroblast reticular cell-lined (FRC) conduits and are sampled by resident DCs (3). Both the packing of collagen fibers within FRC-conduits and direct filtration by lymphatic endothelial cells (LEC)-lining the lymphatic sinus floor determine LN size exclusion properties and thus dictate antigen delivery (3, 4). While lymph flow is constitutive at steady state, lymphatic fluid transport is rapidly reduced following cutaneous infection, indicating that peripheral tissue context and lymphatic vessel function dictate antigen delivery (5).

Within LNs, non-hematopoietic stromal cells generate and maintain chemokine gradients to direct leukocyte recruitment and positioning. Afferent lymphatic vessels direct DC homing and express adhesion molecules that permit transendothelial migration, while specialized blood vessels, high endothelial venules, facilitate naïve lymphocyte entry. FRCs provide a physical scaffold within the paracortex, express homeostatic chemokines that bring mature, antigen-loaded DCs in close proximity with naive T cells (6), and modulate their contractile phenotype to permit LN enlargement and lymphocyte expansion (7). Following activation, T cells egress LNs along shingosine-1-phosphate (S1P) gradients actively maintained by efferent LECs and ultimately recirculate into blood (8). These newly T cell receptor (TCR)-stimulated effector T cells are now proficient to recognize inflamed blood endothelium in peripheral, non-lymphoid tissues and are restricted from re-entry into LNs (9). Importantly, while naïve T cells require TCR stimulation in lymphoid organs for activation, pre-existing memory T cells acquire tissue-homing capability independent of TCR-stimulation and are rapidly mobilized to sites of inflammation where they exert their protective function (5, 10, 11). Thus, though effector and memory cells are subject to the same peripheral tissue microenvironments and barriers upon arrival, the signals required to activate mechanisms of homing and tissue adaptation may be distinct (9).

Just as in secondary lymphoid organs, non-hematopoietic cells in peripheral, non-lymphoid tissue provide a functional scaffold that determines T cell infiltration, motility, effector function, and retention. Tissue remodeling in chronic diseases, such as cancer, significantly alters requirements for T cell behavior and function. Here we discuss the current state of knowledge regarding interaction between T cells and non-hematopoietic stromal components in peripheral, non-lymphoid tissue. How effector and memory T cells adapt within and navigate through these non-hematopoietic barriers is poorly understood, and yet the heterogeneity of tissue structure and function within which T cells impart immune control must necessitate an array of adaptive mechanisms. A more detailed understanding of mechanisms used by effector and memory T cells to adapt to their peripheral tissue environment will provide crucial insight into the ways in which solid tumors inhibit T cell function and mediate immune escape.

## Getting in: T cell extravasation across the vascular endothelium

### Inflamed endothelial cells provide signal two for tissue infiltration

**Figure 1:**
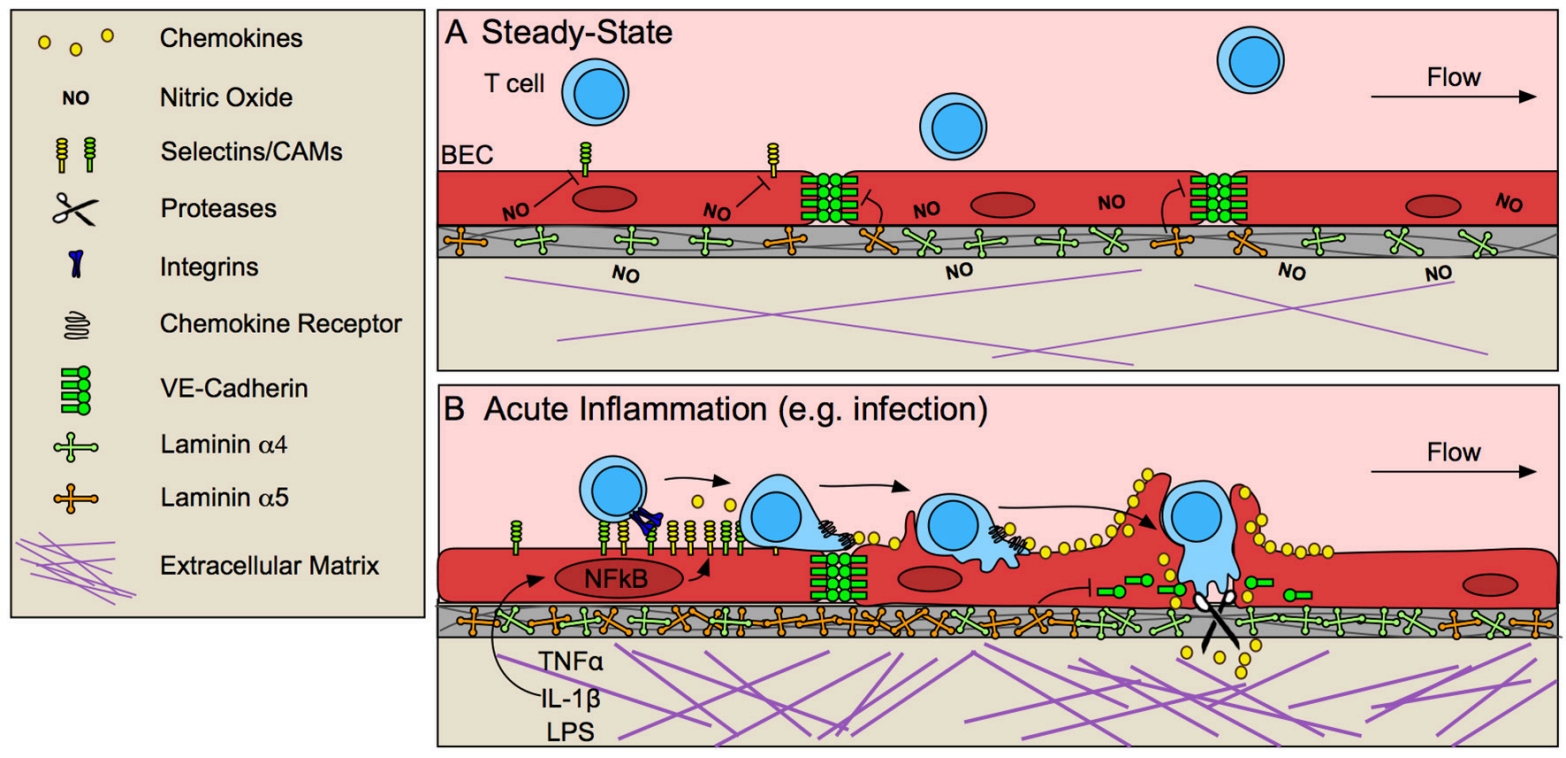
Blood endothelial cells control T cell entry into inflamed tissue. **(A)** The vascular endothelium limits T cell infiltration at steady-state by low expression of selectins and cell adhesion molecules (CAMs), and stabilized endothelial cell-cell junctions, due in part to tonic nitric oxide (NO) signaling and laminin α5-mediated VE-cadherin stabilization. **(B)** In response to pathological challenge and inflammatory stimulus (e.g., TNFα, IL-1β, LPS), blood endothelial cells (BECs) become activated and increase expression of selectins, CAMs and chemokines, which promote lymphocyte adhesion and migration to sites permissible for transmigration. In some cases, BECs form a transmigratory cup that provides a perpendicular scaffold to direct T cell transmigration. Inflammatory remodeling of the basement membrane contributes to lymphocyte access through destabilization of VE-cadherin at endothelial junctions and by generating low-density sites permissive to lymphocyte migration.

Though activated effector and memory T cells acquire the machinery necessary for homing to inflamed tissue in response to TCR and inflammatory stimuli in circulation and lymphoid organs (9), activated vascular endothelial cells (EC) that line post capillary venules in tissue provide the critical signal 2 necessary for infiltration. Lymphocytes home to sites of inflammation following a cascade of adhesive and signaling events mediated by sequential ligation and activation of selectins, integrins, and chemokines on ECs. EC activation and expression of these necessary adhesive molecules occurs only at sites of inflammation, thus ensuring specific infiltration of inflamed tissue (12) and sparing normal, uninflamed tissues from unnecessary lymphocyte infiltration, such that ECs act as key determinants for the anatomic tissue distribution of stimulated lymphocytes (Figure 1).

At steady-state, low levels of lymphocyte adhesion molecule expression (13, 14) is maintained by tonic nitric oxide signaling (15) and lack of inflammatory stimuli. In response to challenge, tissue-resident macrophages, mast cells, and damaged fibroblasts (16) produce tumor necrosis factor α (TNFα) and interleukin-1 (IL-1) (17, 18), which are sufficient to activate local but not systemic ECs (19). Activation of nuclear factor-κB (NF-κB) in ECs by these inflammatory stimuli upregulates P- and E-selectins, intracellular adhesion molecule 1 (ICAM-1), vascular cell adhesion protein 1 (VCAM-1), and chemokines, and EC-specific loss of NF-κB is sufficient to prevent lymphocyte infiltration into tissue (17). Selectins bind to carbohydrate moieties on glycoproteins expressed by effector and memory T cells (9). Selectin binding initiates T cell rolling along the inflamed endothelium (20), allowing for subsequent chemokine detection. Chemokines produced by ECs then direct actin-dependent spreading, polarization, and lateral migration of arrested lymphocytes across the endothelial surface, presumably to identify sites permissive to transmigration, marked by clustered cell adhesion molecule (CAM) expression. High-affinity adhesive interactions between ICAM-1 and VCAM-1 and their respective integrins (LFA-1/αLβ2, and VLA-4/α4β1) ultimately lead to lymphocyte arrest (18).

While the endothelium rapidly responds to inflammatory cues to recruit circulating lymphocytes, it may also inhibit T cell adhesion and migration under certain conditions. T cells have decreased adhesion to inflamed ECs co-cultured with dermal fibroblasts, but not fibroblasts isolated from synovial joints of rheumatoid arthritis patients (21), indicating that fibroblasts help to maintain the endothelial barrier to lymphocyte infiltration in healthy tissue while their dysfunction may promote disease. PEPITEM, a small peptide released from adiponectin-stimulated B cells, binds to cadherin-15 on ECs and triggers production and release of sphingosine 1 phosphate (S1P), which reduces T cell trafficking across endothelium (22), and low expression of adiponectin receptor on B cells is associated with chronic lymphocyte infiltration in diseases such as type 1 diabetes, rheumatoid arthritis, and aging (22).

Upon adhesion to inflamed endothelium, lymphocytes next traverse the endothelial barrier. Endothelial cells actively support and guide lymphocytes to sites permissive to transmigration while still maintaining barrier integrity via integrin-dependent mechanisms of actin remodeling (23). At sites of transmigration, ICAM-1/LFA-1 and VCAM-1/VLA-4 clustering forms an immunological synapse-like interaction between ECs and T cells (24), concentrating adhesion molecules into a ring structure (25). ECs often extend microvilli symmetrically around T cells to form a “transmigratory cup” (26) which further strengthens adhesion and provides a perpendicular scaffold to promote transmigration (24). Ultimately, T cells pass through the endothelium in one of two ways, either between ECs at intercellular junctions (paracellular route), or directly through individual ECs (transcellular route). Transcellular migration seems to be initiated by invadosome-like protrusions on lymphocytes (27). Paracellular migration, on the other hand, requires EC-mediated destabilization of vascular endothelial cadherin (VE-cadherin) at endothelial cell-cell junctions (28) and is further mediated by integrins, CAMs, and other adhesion molecules such as PECAM-1, JAM-1, and CD99 (18).

Destabilization of VE-cadherin at EC cell-cell junctions seems to be necessary for lymphocyte transmigration (29). ECs expressing a mutant form of VE-cadherin that is not endocytosed and therefore retained at cell-cell junctions, prevents lymphocyte recruitment to inflamed skin (28). Blockade of VE-cadherin stabilizing integrins, β1 and β3 (29) or dephosphorylation of tyrosine 731 by SHP-2 targets VE-cadherin for endocytosis and subsequently increases neutrophil transmigration *in vitro* (30). Interestingly, lymphocyte binding to ECs induces SHP-2-mediated VE-cadherin destabilization (30), indicating that lymphocyte adhesion may prime ECs to be permissive of transmigration. VE-cadherin is also cleaved by a disintegrin and metalloproteinase 10 (ADAM10) and tetrospanin 5 and 17, expressed by inflamed ECs, and EC-specific loss of ADAM-10 delays T cell, but not neutrophil or B cell, transmigration *in vitro* (31). Interestingly, proteolytically active leukocytes, such as neutrophils, may mediate cleavage necessary for lymphocyte transmigration in the absence of EC proteolysis (31). Thus, even if not intrinsically proteolytic, leukocyte protease activity may positively promote lymphocyte transmigration across inflamed endothelium *in vivo*.

### Antigen-dependence of T cell recruitment and extravasation

Peripheral effector (12) and memory T cells (11) are recruited to inflamed tissue in an antigen-independent manner, indicating that local presentation of cognate antigen is not necessary for tissue infiltration. The antigen-independence of T cell recruitment is exemplified by recent studies that demonstrate abundant bystander, pathogen-specific T cells, in solid tumors (32). Interestingly, however, homing of insulin-specific CD8^+^ T cells to pancreatic islets, but not other tissues, is reduced with loss of major histocompatibility complex class I (MHC-I) *in vivo* (33) and antigen-loaded MHC-I presented on luminal surfaces of the blood-brain barrier was functionally required for antigen-specific T cell trafficking to the brain (34). These observations have led to the hypothesis that antigen presentation by ECs may amplify antigen-specific T cell recruitment in certain tissues and disease states. ECs dynamically express MHC-I and MHC-II during inflammatory processes and possess antigen-processing machinery necessary for cross-presentation of exogenous antigens (35). Human ECs scavenge and cross-present the type I diabetes islet autoantigen GAD65 on MHC-II and this enhances the transmigration of antigen-specific T cells *in vitro* (36). Further *in vitro* evidence supports both inhibitory (37) and promotional (38, 39) roles for EC antigen presentation in lymphocyte trafficking, indicating that antigen-presented by ECs may provide context-dependent “go” or “stop” signals that tune T cell infiltration.

Interestingly, ECs express a variety of T cell costimulatory and coinhibitory molecules (24), and as such, may represent semi-professional APCs strategically placed to interact with activated effector and memory T cell populations. In addition to tuning transmigration, data from various tissues indicate that ECs may employ their repertoire of immune checkpoints and APC-like function to mediate peripheral tolerance and modify T cell behavior as they transmigrate or arrest at the vascular interface. For example, liver sinusoidal endothelial cells scavenge and cross-present food-borne antigens and induce tolerance through T cell adhesion and sequestration in the liver (40, 41), and tumor-associated LECs cross-present exogenous antigens (42, 43) and maintain peripheral tolerance to self-antigens in LNs (44, 45) dependent on constitutive expression of programmed death-ligand 1 (PD-L1) (45). The relative significance of EC antigen presentation *in vivo*, however, is likely both tissue and disease specific. Further testing is needed and specifically, EC-specific knockdown strategies, to determine the functional relevance of EC antigen-processing and presentation *in vivo*.

### Overcoming the basement membrane

The final and rate-limiting step in lymphocyte extravasation is crossing the basement membrane (46). The basement membrane is a 20–200 nm thick dense proteinaceous substrate composed of laminins, collagen type IV, and sulfated proteoglycans (47), that separates the vascular endothelium from extracellular matrix (ECM) in the tissue parenchyma. Laminins and collagen IV produced by ECs self-assemble into a dense sheet that is crosslinked by perlecans and nidogen and contains 2–5 μm-in-diameter pore-like regions of low protein density (48), presumed sites of lymphocyte passage. Basement membrane composition differs between developmental stage, vessel type, and activation state of the endothelium (47), with particular variability of laminin isoforms. Laminins are composed of alpha, beta, and gamma chains (e.g., laminin α4, β1, γ1 is denoted as laminin 411), and presence in basement membrane is context and location dependent. In the central nervous system (CNS), laminin α4 is ubiquitous (49), while laminin α5 expression is patchy and irregular (50), but both are increased upon inflammation (47, 51). In murine experimental autoimmune encephalomyelitis extravasation occurs predominantly at sites of low laminin α5 density (29, 51) and laminin α5 is sufficient to inhibit T cell transmigration in a dose-dependent manner *in vitro* (50). Additionally, laminin α4-deficient mice increase expression of laminin α5 in the CNS leading to decreased T cell migration across the blood brain barrier in EAE (50), suggesting that the composition of laminins in the basement membrane may selectively regulate T cell transmigration.

The mechanisms by which different laminin isoforms regulate T cell transmigration are unclear. Laminin α5 binds to integrin β1 and β3 on ECs and stabilizes VE-cadherin at EC junctions (29). Activated lymphocytes also express integrin β1 (18), however, and it is possible that laminin α5 may signal directly to infiltrating lymphocytes and instruct transmigration, although this has not been investigated. Regardless of how T cells get across the EC layer, the basement membrane is a dense, proteinaceous barrier that they must penetrate to complete diapedesis. Neutrophils express elastase to remodel regions of low basement membrane density allowing for their tissue infiltration (52), however, the specific mechanisms of lymphocyte migration through the basement membrane is unclear. The small size and pliability of lymphocytes and their nuclei may permit movement through the 2–5 μm pore-like regions of the basement membrane. However, T cell intrinsic loss of granzyme B (GrzB), which degrades both collagenous and non-collagenous ECMs (53, 54), reduces extravasation *in vivo* (54), indicating proteolysis may be required for basement membrane penetration. Further studies are needed to evaluate the contribution of the basement membrane to selective lymphocyte extravasation in acute and chronically inflamed tissue.

## Moving around: T cell interstitial migration in homeostatic and inflamed peripheral tissues

**Figure 2:**
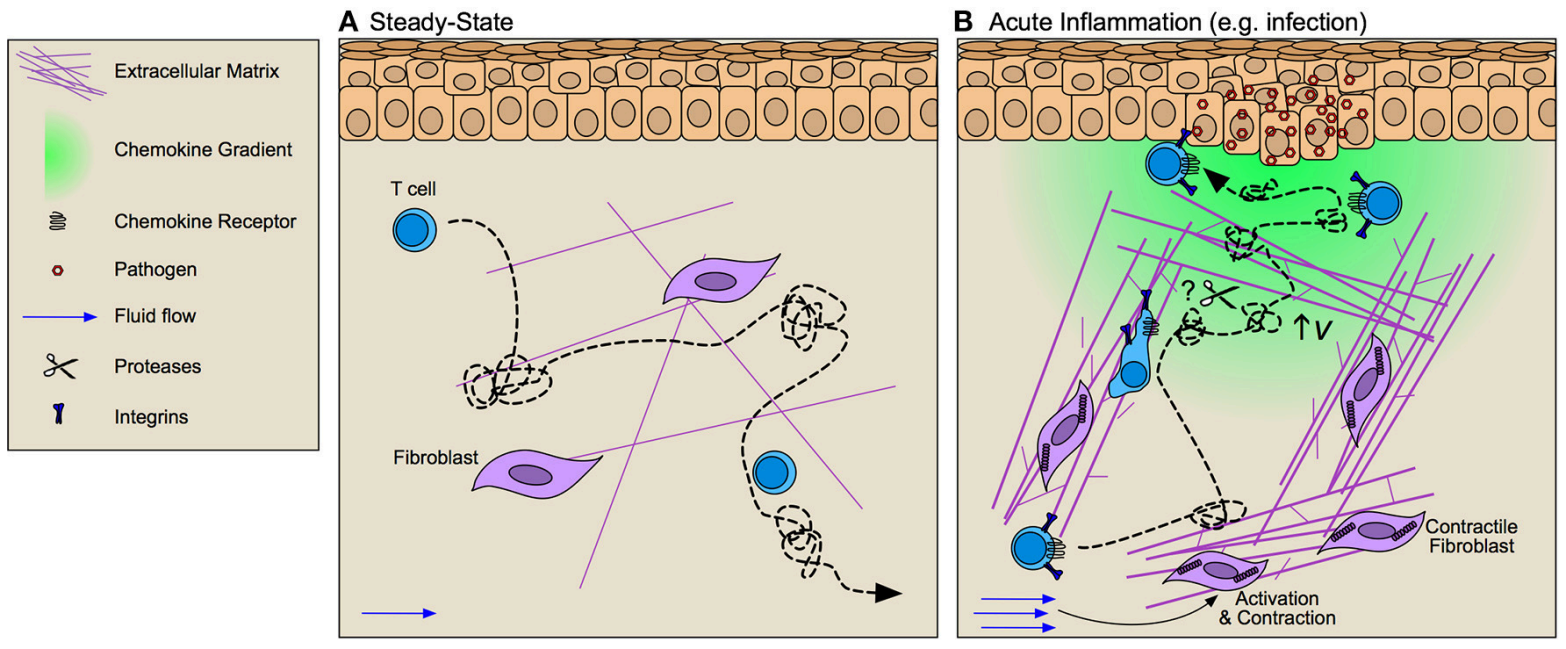
Interstitial matrices control T cell migration through inflamed tissue. **(A)** At steady-state T cells exhibit integrin-independent amoeboid-like Lévy walk behavior (dotted line) that facilitates their surveillance of peripheral tissue. **(B)** During inflammation fibroblast activation alters tissue tension through increased deposition, bundling, and cross-linking of extracellular matrix (ECM) components thereby altering the scaffold across which T cells must migrate. Increased interstitial fluid flows that result from vascular permeability further activate fibroblasts and promote directional fiber alignment. These inflammation-induced ECM changes activate integrin-dependent lymphocyte migration along collagen bundles. Whether T cell migration is dependent upon ECM organization or if rather lymphocytes may activate proteolytic activity to promote tissue invasion remains largely unclear. Facilitating T cell position at the site of challenge are chemokine gradients (e.g., CXCL9/10) that increase lymphocyte velocity (v) thereby improving their Lévy walk search efficiency and permitting accumulation at and around target cells.

Following extravasation from the vasculature, effector and memory T cells encounter the complex heterogenous interstitial matrix through which they must traverse and locate target cells (Figure 2). The ECM defines the 3D structure of tissues and exhibits heterogeneity across tissue types and disease states. The topography of the interstitial matrix is determined by the combination of structural and non-structural glycoproteins such as fibrillar collagen, elastin, fibronectin, laminin, and tenascin, decorated by associating proteoglycans (e.g., decorin and versican), which contain glycosaminoglycans (GAGs; e.g., heparan sulfate and chondroitin sulfate). The physical spacing and orientation of fibrillar proteins, as well as net charge of decorating GAGs, determines matrix porosity, rigidity, and bioactive molecule presentation. Both the physical and molecular properties of the ECM determine necessary modes of leukocyte migration and thus the efficiency with which leukocytes survey tissue. Compared to innate immune cells (e.g., neutrophil and DC), the mechanisms that govern the interstitial motility and homing behavior of T cells are poorly defined, yet adaptation to and utilization of chemical and physical signals in heterogenous tissues is necessary to rapidly identify rare APCs and mediate their local effector function. Tissue biophysics, matrix rigidity and interstitial fluid flux, may be an important control point for tissue T cell dysfunction. Whether T cell motility is simply a function of the existing microenvironment, or if rather T cells may exert force within interstitial tissues to direct their movement is critical to understanding diseases where T cell infiltration is impaired, such as cancer.

### Distinct mechanisms of T cell interstitial migration in naïve and inflamed tissue

Molecular mechanisms of T cell interstitial migration (55, 56) have largely been determined in the context of 2D and 3D *in vitro* experimental systems that allow for control of physical and chemical cues to determine specific effects on T cell motility. These studies indicate that in naïve matrices lymphocytes primarily utilize amoeboid modes of movement independent of focalized adhesions and pericellular proteolysis (57, 58). Nuclear squeezing and deformation permits lymphocyte movement along preformed structures at velocities 10–40 fold higher than adhesion-dependent, mesenchymal migration (59). These studies have importantly established guiding principles for understanding T cell behavior in 3D, however, fail to capture the full complexity of inflamed interstitial matrices, which exhibit altered collagen fiber density, orientation, and composition relative to naïve in a tissue-specific and challenge-specific manner. Highlighting the discrepancies found between naïve matrices *in vitro* and inflamed tissue *in vivo*, intravital imaging in inflamed skin indicate that Th1 T cell motility is integrin-dependent, where specifically αv integrins (paired either with β1 or β3) facilitate T cell motility along matrix fibers (60). Matrix remodeling, therefore, necessitates T cell adaptation and activation of adhesion-dependent modes of migration. Importantly, effector and memory T cells do express an array of matrix-binding integrins that increase in expression upon activation and provide ligand specificity for various matrix components (61). Furthermore, T cells extracted from gut are more adhesive to ECM *in vitro* than circulating lymphocytes (62) and CD4^+^ effector T cells display distinct integrin repertoires when extracted from skin or lung, consistent with the differences of ECM composition in each tissue (60). Whether T cells tune their integrin repertoire in the context of their resident ECM, are imprinted at priming to prefer certain matrices, or a combination of both, remains an open question.

Interestingly, intravital imaging largely demonstrates that T cells preferentially follow pre-formed networks of fibrillar structures (55, 60, 63). 3D confinement studies *in vitro* indicate that limiting the space through which T cells can migrate significantly alters migratory speeds (64) and thus within a heterogenous interstitial matrix, it has been proposed that T cells may simply follow the path of least resistance (65). While in healthy tissue, pre-formed ECM tracks may facilitate the directional migration of T cells toward their target, under pathological conditions such as fibrosis and cancer, increased ECM density and rigidity (66) may act as a barrier to T cell infiltration and motility (discussed in more detail later). Consistent with this hypothesis, dynamic imaging reveals that matrix fiber density and orientation at tumor borders directs peripheral but not intratumoral T cell motility (63), and liver fibrosis inhibits T cell-mediated killing of infected hepatocytes (67), suggesting that T cells are incapable of proteolytically invading dense matrix structures.

Peripheral T cells, however, do express a limited array of proteases upon antigen stimulation and cytokine exposure. Matrix metalloproteinase 2 (MMP-2) is upregulated following VCAM-dependent adhesion to ECs (68), likely facilitating T lymphocyte invasion across the basement membrane. Inflammatory mediators [e.g., CCL5, prostaglandin E2, leukotriene B4 (LTB4), TNFα, transforming growth factor β (TGFβ), and IL-2] induce MMP-9 expression while type I and II interferons (IFN) are suppressive (68). Granzyme B, secreted by activated T cells, has protease activity (53, 54) and urokinase degrades laminins and fibronectin and activates some latent form of MMPs (69). Importantly, evidence for protease-dependent tissue invasion of T cells was identified in rheumatoid arthritis patients. T cells isolated from peripheral blood of rheumatoid arthritis patients display increased invasive capacity *in vitro* and *in vivo* dependent upon elevated expression Tks5, a scaffold protein required for the formation of matrix-degrading invadopodia (70). Thus, at least in some diseased states, T cells may activate intrinsic proteolysis that supports their pathologic activity.

While the ECM provides an instructional scaffold upon which leukocytes migrate through the interstitium, diffuse matrix-bound chemokine gradients are proposed to determine positioning and directionality. Intravital imaging, however, fails to identify persistent and directional T cell homing *in vivo* and T cells rather exhibit a Lévy walk motility pattern characterized by straight runs at fixed velocity interspersed with pauses (71). Interestingly, blockade of the CXCR3 ligand, CXCL10, in a model of *Toxoplasma* infection of CNS resulted in reduced CD8^+^ T cell velocity but did not alter Lévy walk patterns, indicating that CXCL10 may function to improve protective immunity by increasing T cell speed rather than through directional migration (71). Thus, reduced velocity impairs searching efficiency and reduces lymphocyte recruitment to and positioning at infectious foci. Consistently, CXCR3 expression on T cells was dispensable for vascular extravasation but required for localization to infected foci in cutaneous vaccinia infection (72). Continued understanding of the ways in which chemokines affect T lymphocyte behavior in tissues requires further intravital imaging studies to directly assay the dynamic behavior of T cells in the presence or absence of specific chemoattractant and chemorepellant molecules.

### Organization of the interstitial matrix by fluid flows

Constitutive, interstitial fluid flows are kept in constant motion by hydrostatic and osmotic pressure differences between blood vessels, the interstitium, and lymphatic vessels (73). Interstitial fluid flow is slow, ranging from 0.1–2 μm/s at steady state and increased during inflammation and in tumors (73). Lymphatic vessels dynamically regulate fluid transport and consequently influence levels of interstitial fluid flow, which may in turn affect interstitial immunity. Cutaneous lymphatic vessels rapidly shut down fluid transport in a type I IFN-dependent manner preventing passive viral dissemination to LNs (5). Furthermore, mosquito saliva is sufficient to induce local edema and an inflammatory influx of neutrophils that when delivered in combination with virus, promotes viral retention at the bite site and enhances infection (74). Thus, modulation of fluid transport phenomena (either through enhanced vascular leakiness or altered lymphatic transport) may be a critical feature of tissue infection, which remains to be explored more carefully.

Dynamic regulation of fluid flux through tissue impacts ECM density, stiffness, and alignment and thus the scaffold within which cell motility is directed. Increased interstitial fluid pressure activates fibroblasts through integrin signaling and TGF-β upregulation of α-smooth muscle actin (αSMA) thereby increasing fibroblast contractility and subsequent alignment and bundling of collagen fibers (75). Flow additionally influences the expression and distribution of soluble and matrix bound factors along this scaffold to inform interstitial cell motility. Pericellular gradients can be established via multiple mechanisms including proteolytic release from ECM, degradation, metabolism, or removal by decoy receptors of a local source of attractants (76). Additionally, interstitial fluid flow introduces directional bias in chemokine distribution when expressed by migrating cells setting up functional gradients in the direction of flow. Termed autologous chemotaxis, this mechanism may support directional migration of tumor cells (77) and DCs to draining peripheral lymphatic vessels. How interstitial fluid flows influence T cell motility either directly, or indirectly remains to be experimentally tested.

## Getting out: T cell egress via lymphatic vessels

### Lymphocytes exit peripheral, non-lymphoid tissues

**Figure 3:**
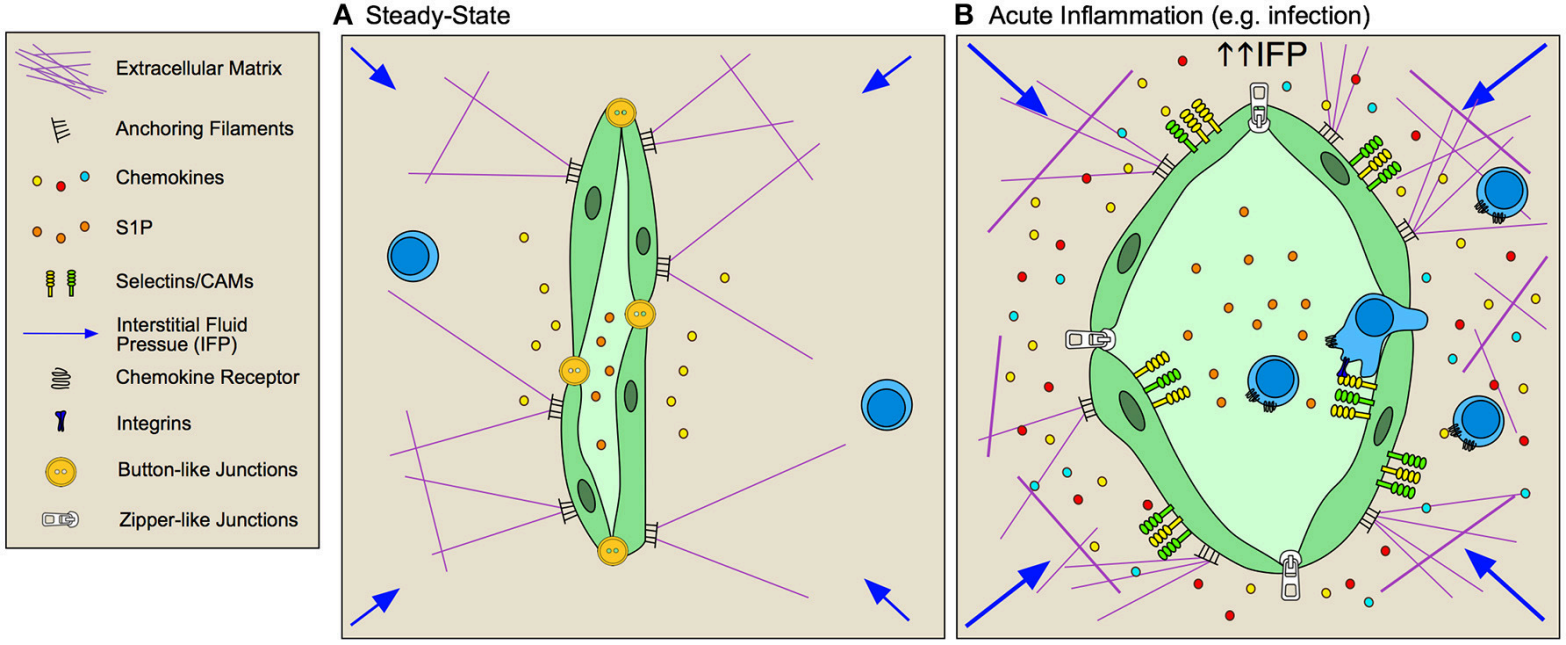
Inflamed lymphatic vessels promote lymphocyte exit from tissue. **(A)** Steady state lymphatic endothelial cells (LEC) form loose button-like junctions in lymphatic capillaries, and constitutively transport interstitial fluid from peripheral non-lymphoid tissue to draining lymph nodes. Production of homoestatic chemokines, such as CCL21, supports immune surveillance by directing leukocyte homing toward lymphatic vessels and tissue egress. **(B)** During inflammation, lymphatic vessels respond to both biochemical and biophysical stimuli to adapt their function within the tissue. Lymphatic vessels are activated by inflammatory cytokines and elevated interstitial fluid flow resulting in increased expression of selectins, cell adhesion molecules (CAMs) and context-dependent chemokine secretion that together promotes lymphocyte egress from inflamed tissues. LECs remodel their junctions, going from loose, button-like junctions to tight, zipper-like junctions, which is associated with decreased fluid transport from inflamed tissues and subsequent increased interstitial fluid pressures.

Following entry into and surveillance of tissue at least a subset of T cells continue on and egress out through lymphatic vessels (Figure 3). Parabiosis experiments demonstrate that most endogenous memory T cells in peripheral tissue reach equilibrium with migratory blood-borne donor T cells indicating rapid turnover in peripheral tissue (78) [with the notable exception of resident memory lymphocytes 79). In sheep, where lymph can be readily sampled, afferent lymph contains 10^6^ cells/ml (80). Furthermore, the number of leukocytes in lymph are increased by sometimes as much as 100 fold during acute and chronic inflammatory signals (80), indicating tissue egress is influenced by context. Whether the cellular component of afferent lymph is simply a reflection of the tissue it drains (e.g., passive, random transport) or rather represents a subset of tissue lymphocytes (e.g., active, selective transport) remains largely unknown.

At least two candidate molecules have been proposed as necessary signals for lymphatic directed egress, CCL21/CCR7 and S1P/S1PR1. In models of acute lung and skin infection, T cells egress from inflamed peripheral tissue in a CCR7-dependent manner (81, 82), such that CD4^+^ T cells accumulate in epithelial tissue of CCR7^−/−^ mice in an age-dependent manner (83). Additionally, forced overexpression of the spingosine-1-phosphate receptor (S1PR1) prevents the establishment of tissue resident memory CD8^+^ T cells, suggesting that the inability to respond to S1P gradients maintained by lymphatic vessels is necessary for local retention (84). Treatment with FTY720 (S1PR1 agonist) only partially inhibits egress (85), but does improve CD69-deficient CD8^+^ T cell persistence in skin after HSV infection (86). Thus, this together with low levels of CCR7 and expression of the E-cadherin binding CD103 and β1 integrin may promote retention (12, 87, 88). Conversely, egress from chronically inflamed tissue is pertussis toxin sensitive but CCR7-independent (85), indicating a role for alternative G-protein coupled receptors. CXCR4 might represent an alternative mechanism of lymphatic vessel directed egress. DC trafficking is reduced but not completely eliminated in CCR7^−/−^ mice, and inhibition of CXCR4 further reduced DC trafficking to draining LNs in a model of contact hypersensitivity (89), but evidence for CXCR4-dependent T cell egress is lacking (90). Studies using quantitative models that track endogenous populations of tissue-resident lymphocytes, either through intravital imaging or photoconvertible mice, remain necessary to provide clarity regarding the molecular mechanisms that determine the context-dependence and specificity of leukocyte egress from inflamed tissues.

### Lymphatic vessels regulate tissue exit

To facilitate tissue exit, lymphatic vessels express an array of chemokines in a context-dependent manner. LECs constitutively express CCL21 (91, 92) and further increase expression during chronic lung inflammation (90) and acute inflammation in skin, but not treatment with complete Freund's adjuvent (CFA) (93). TNFα stimulation of LECs causes release of CCL21 stores (94) and *de novo* production of CCL21 (94) as well as a host of other chemokines including CCL20, CXCL5, CCL5, CXCL2, CX_3_CL1, and CCL2 (95). Additionally, *in vitro* analysis indicates that lipoteichoic acid, a component of gram-positive bacterial cell walls, induces TLR2-dependent expression of CXCL1, CXCL3, CXCL6, and CXCL8 (96). *In vivo* analysis of mRNA from LECs in inflamed skin confirms these *in vitro* results, and also identified several other chemokines expressed by LECs, including the CD8^+^ T cell-homing chemokines CXCL9 and CXCL10 (93), all together indicating that the chemokine repertoire produced by LECs in peripheral tissue is context dependent. Consequently, how this diverse repertoire of chemokines produced by inflamed LECs functionally regulates lymphocyte egress from tissue remains a largely open question.

LECs, like BECs, increase expression of the T cell adhesion molecules in response to local inflammation and interstitial fluid flows. ICAM, VCAM, and E-selectin are expressed on the LEC surface rapidly following peripheral challenge *in vivo* (5, 93, 95) and following stimulation *in vitro* (97). LFA-1 is necessary for naïve T cell egress from inflamed skin (98) and inhibition of vascular endothelial receptor-1 (CLEVER-1) and macrophage mannose receptor prevented T cell migration through afferent lymphatic vessels to draining LNs (99, 100). The requirement for integrins in LEC transendothelial migration in inflamed tissue may mirror the differential integrin requirement for DCs. While DCs in skin squeeze through overlapping, button-like junctions in naïve lymphatic capillaries (101), transmigration across inflamed vessels requires integrin-mediated adhesion (97). Interestingly, cutaneous viral infection (5) and tracheal bacterial infection (102) induces lymphatic capillary remodeling of naïve button-like junctions to tight, zipper-junctions, typically found in deeper collecting vessels. These reversible changes may generate a less permeable endothelium and thus determine the integrin dependence of cellular transport. The functional relevance of lymphocyte egress at both steady-state and during inflammation remains to be determined, and in particular whether lymphocytes exit tissue to mediate immune resolution or rather enter LNs for re-stimulation by professional APCs remains an open and interesting question.

## Adaptation in tumor microenvironments

**Figure 4:**
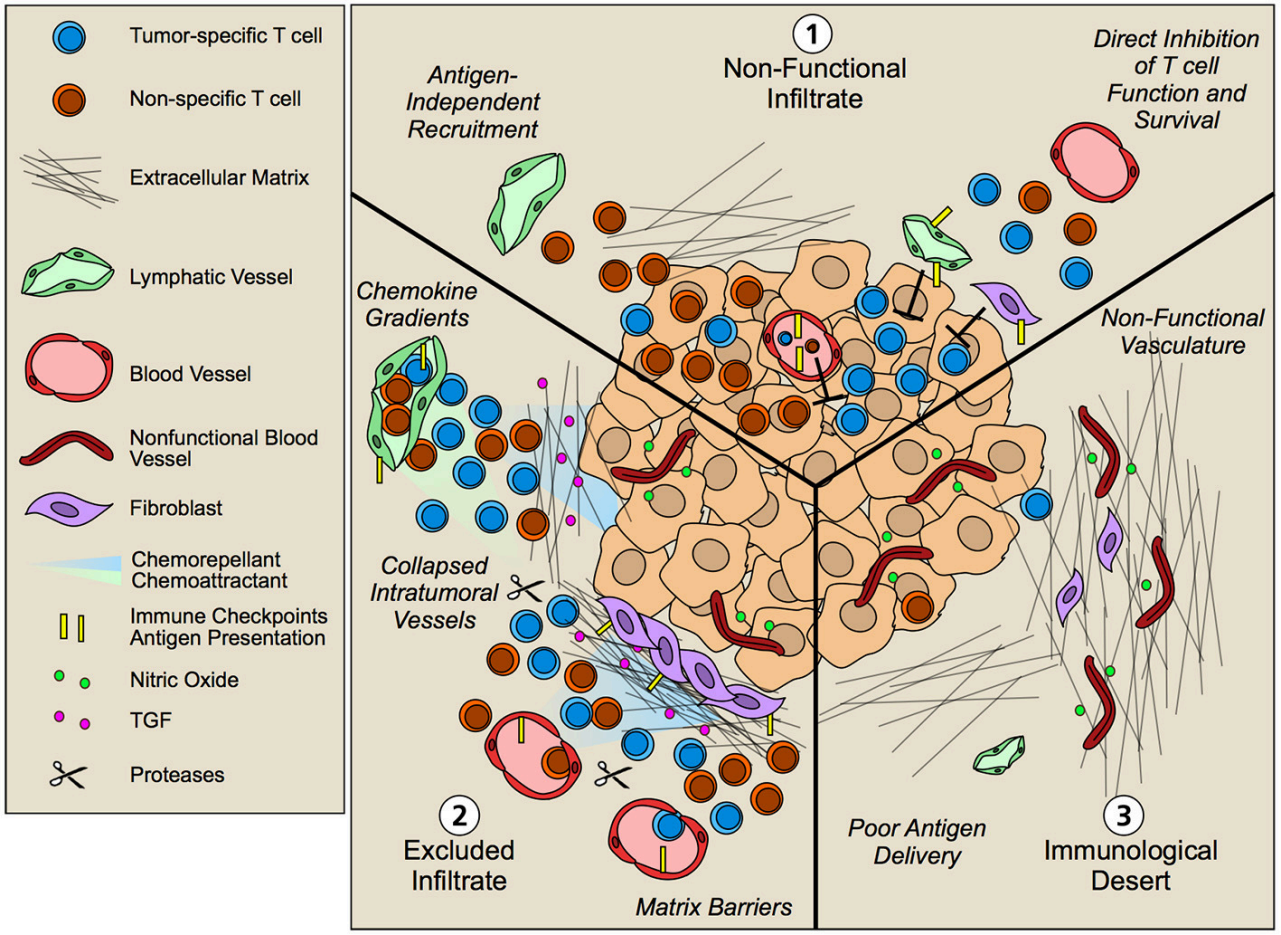
Non-hematopoietic cell contribution to tumor immune landscapes. The geographic distribution of T cells within intratumoral and peritumoral regions is both predictive of overall survival and response to immunotherapy. Patients that fail to respond to immunotherapy often exhibit three patterns of T cell infiltrate that are governed by an array of mechanisms including contributions from tumor cells and infiltrating hematopoietic cells. Non-hematopoetic cells, however, additionally contribute to the infiltration, retention, and function of T lymphocytes in tumor microenvironments. **(1)** Non-functional infiltrate: possessing an intratumoral but seemingly ineffective infiltrate. Antigen-independent recruitment of both effector and memory T cells subsets by vascular endothelium generates a diverse repertoire of T cells both relevant and irrelevant for tumor killing. Upon tissue entry, non-hematopoietic cells further exert multiple mechanisms of immune suppression, including expression of immune checkpoints such as PD-L1 and FasL that limit local T cell function. **(2)** Excluded infiltrate: possessing a T cell infiltrate that is restricted to the tumor periphery. Establishment of matrix barriers, collapsed intratumoral vessels, poor expression of adhesion molecules, and collaborating chemoattractant and chemorepellant gradients likely all contribute to the exclusion of T cells at the periphery of tumor nests such that inhibition of these features may improve infiltration. **(3)** Immunological desert: Completely lacking a T cell infiltrate in both tumor nests and stroma. Impaired lymphatic transport may result in poor antigen delivery to lymph nodes and thus poor priming. However, even in the presence of an activated systemic T cell pool, non-functional vessels driven by the angiogenic and desmoplastic tumor microenvironment may prevent local infiltration leading to lesion-specific differences in immune infiltrates.

Though tumors were previously thought to be poorly immunogenic and not capable of activating an immune response, we now know that somatic mutations (generated by DNA instability and environmental challenge) generate neo-antigens that are sufficiently distinct from self, such that T cells are capable of expanding and directing tumor-specific killing. Thus, the accumulation of neo-antigens in tumors is likely a prerequisite to anti-tumor immunity across tumor types, and consistently, those tumors that exhibit highest somatic mutational burden, e.g., melanoma, exhibit good overall response to immune checkpoint blockade (103). Even in the presence of potent neo-antigens, however, some tumors still fail to respond to therapy and somatic mutational burden is not sufficient to predict T cell infiltration within and across tumor types (104). Thus, multiple overlapping mechanisms of immune suppression create a more complex immune landscape, such that, as discussed above, processes of T cell recruitment, retention, survival, and exit may underscore intratumoral T cell presence and thus influence response to therapy. Current efforts to define biomarkers that are predictive of response to immune checkpoint blockade reveal an array of factors from myeloid cells to the microbiome, that affect patient response. Here we will focus on the localization of T cells within and around the tumor parenchyma as one indicator of responsiveness and discuss how tumor-induced stromal remodeling may contribute to T cell distribution (Figure 4).

Multiple studies across tumor types now indicate that the presence of T cells within tumor nests is predictive of response to therapy (105). As a consequence, non-responding tumors typically exhibit T cell infiltrates that are described by three main patterns: (1) non-functional immune responses, possessing an intratumoral but seemingly ineffective infiltrate; (2) tissue excluded T cell infiltrates, possessing a T cell infiltrate that is restricted to the tumor periphery; and (3) immunological deserts, completely lacking T cell infiltrate both in the tumor nests and in adjacent stromal (106). The underlying biology that regulates these patterns of T cell infiltration is clearly multifactorial—some of the contributing factors from the perspective of the non-hematopoietic tumor stroma are discussed below.

### Non-functional T cell infiltrates

Non-functional immune infiltrates (106), refers to tumors containing intratumoral lymphocytes in both pre- and post-therapy biopsies that do not contribute to significant clinical response. Importantly, methods to evaluate intratumoral T cell populations largely quantify changes in bulk T cell populations (CD4 or CD8), and even when enriched for markers of previous antigen exposure (CD45RO) or effector function (GrzB) likely still quantify a heterogenous pool of effector, effector memory, and central memory T cells that represent a range of antigen specificities both relevant and irrelevant to the tumor. Rapid recruitment of effector and memory T cells is antigen-independent (11, 12), and bystander, viral-specific T cells (e.g., HCMV or EBV-specific) are abundant in human tumor tissue (32). Thus, efforts to specifically quantify tumor-reactive T cell clones may be more predictive than bulk T cell populations. Consistent with this hypothesis, CD39 was recently identified as a marker to distinguish tumor antigen-specific CD8^+^ T cells from bystander T cells across multiple tumor types (32, 107) and stratification of patients based on frequency of CD39^+^CD103^+^ double positive CD8^+^ T cells associated with increased overall survival in head and neck cancer patients (107). Thus, because of the promiscuity of T cell infiltration across the vascular endothelium, the presence of bulk T cells in tumor microenvironments may be insufficient to indicate response. Even when tumors are well infiltrated with antigen-specific T cells, however, multiple additional mechanisms suppress their local effector function mediated by tumor, hematopoietic (108), and non-hematopoietic stromal cells.

Aberrant tumor angiogenesis and disrupted fluid flows in tumor microenvironments generate hypoxia and increased interstitial fluid pressures in solid tumors (73, 109) that influence T cell function. Hypoxia induces Warburg effect by cancer cells, leading to increased acidification and lactate production, both of which inhibit cytotoxic activity of lymphocytes *in vivo* (110, 111). Furthermore, increased interstitial fluid flow in the tumor microenvironment activates fibroblasts leading to TGFβ production (73) and ECM contraction. ECM contraction together with shear stress activates stromal stores of latent TGFβ (112), which attenuates CD8^+^ T cell cytotoxicity (113) making them non-responsive to TCR signaling (114). Thus, the disrupted fluid mechanics within tumor tissue may itself participate in the regulation of local T cell function.

Furthermore, non-hematopoietic cells likely exert direct effects on T cells within tumor microenvironments. In the LN, LECs, and FRCs display specific immunological properties that function to maintain peripheral tolerance at steady state, and while we have drawn parallels between the structural role of LN stromal cells and non-hematopoietic cells in peripheral, non-lymphoid tissues, it remains less clear whether peripheral non-hematopoietic cells also acquire immunomodulatory properties characteristic of LN stroma. LN LECs express peripheral tissue antigens in an Aire-independent manner (44, 115); can scavenge and cross present exogenous antigens leading to dysfunctional CD8^+^ T cell activation (42, 43); and can receive peptide-MHC-II expressing exosomes from DCs and induce CD4^+^ T cell hyporesponsiveness (116). In tumors LECs are also capable of scavenging tumor-associated antigens and cross-presenting them on MHC-I (42), however, whether LEC antigen presentation functionally contributes to peripheral T cell responses remains unknown. LECs further are capable of inhibiting DC maturation and function (117) and T cell proliferation through the production of nitric oxide (118), demonstrating that LN-resident LECs inhibit T cell activation and proliferation both directly and indirectly. LN LECs also constitutively express PD-L1 and delete naïve, self-reactive CD8^+^ T cells (45) and peripheral BECs and LECs express PD-L1 in tumors (119–121) and infected tissue (121, 122). Loss of non-hematopoietic PD-L1 and inhibition of IFNγ signaling on peripheral LECs, thus preventing PD-L1 upregulation, improved the persistence of anti-tumor CD8^+^ T cell-mediated tumor killing and overall survival in melanoma-bearing mice (121). Importantly, loss of IFNγ signaling in LECs also promoted the accumulation of anti-viral T cells in infected skin and exacerbated tissue pathology (121). Thus, tumors may coopt normal non-hematopoietic-based mechanisms of tissue protection for immune escape.

Similarly, FRCs exhibit immunomodulatory function in LNs. FRCs express and present peripheral tissue antigens to T cells in LNs (115), receive peptide-MHC-II loaded exosomes from DCs and induce CD4^+^ T cell hyporesponsiveness (116), and dampen T cell proliferation through the production of nitric oxide (118, 123, 124). Interestingly, some cancer-associated fibroblasts acquire markers consistent with FRCs, namely expression of podoplanin and ER-TR7 (125). Whether these fibroblasts represent a unique subset that exhibits immune suppressive function remains unclear, though a recent study demonstrates that tumor-associated fibroblasts in melanoma models cross-present tumor antigens and inhibit T cells in a FasL and PD-L2 dependent manner (126). In LNs, migrating DCs inhibit FRC contraction through CLEC-2/PDPN interactions leading to scaffold relaxation and more space for accumulating T cells (7). Whether leukocytes directly modulate fibroblast contraction in peripheral tissue remains to be tested but might have important implications for T cell invasion into dense, desmoplastic stroma. Further work is necessary to determine the functional relevance of stromal relative to hematopoietic or tumor-mediated immune suppression.

### Excluded infiltrates

T cell exclusion, in which T cells are absent from tumor nests and rather retained in adjacent, surrounding stroma (106, 127) is a significant barrier to response to therapy. One leading hypothesis is that tissue desmoplasia, the aberrant synthesis, alignment, and crosslinking of ECM proteins by fibroblasts in tumor microenvironments (128, 129), creates a physical barrier that prevents T cell invasion. Pancreatic ductal adenocarcinoma (PDAC) is particularly fibrotic and breast carcinomas exhibit stiff collagen fibers in parallel alignment tangential to tumor borders that correlate with poor prognosis (66, 130). Furthermore, dynamic intravital imaging reveals T cell migration along collagen fibers and vessels in tumors (63, 131), consistent with their preferred amoeboid-like mode of migration described in non-malignant matrices. Thus it has been proposed that the orientation and density of matrix fibers prevents T cell infiltration into tumor parenchyma (63).

However, strategies to reduce fibrosis in mouse PDAC models have had mixed results. Though cancer-associated fibroblasts are attributed an array of tumor-promoting activities, including immune suppression, their bulk depletion in the context of PDAC did not improve tumor control but instead drove more aggressive tumor invasion and metastasis (132, 133). In contrast, strategies that rather target the composition of the ECM using enzymes that degrade specific components (e.g., hyaluronan) thus reducing interstitial fluid pressures (134), or reduce ECM deposition and alignment (135) improved immune infiltration and therapeutic response. Interestingly, CAR T cells have had limited utility in solid tumors at least in part due to physical tissue barriers that prevent their infiltration. Consistent with this hypothesis, CAR T cells expressing heparanase exhibit enhanced tumor infiltration and antitumor function (136). Finally, a TGF-β signature was specifically identified in a subset of PD-L1 inhibitor resistant patients exhibiting an excluded infiltrate (not dysfunctional or desert) where T cells were restricted to the fibroblast- and collagen-rich peritumoral region characteristic of metastatic urothelial carcinoma (137). Therapeutic co-administration of antibodies targeting TGF-β and PD-L1 facilitated T cell penetration into the tumor center and provoked a vigorous adaptive immune response leading to tumor regression in a large Phase II clinical trial testing atezolizumab in patients with metastatic urothelial carcinoma (137). Thus, while the ECM facilitates T cell migration in naïve or acutely inflamed tissue, tumor-associated matrix remodeling may suppress T cell motility or place new requirements for interstitial proteolysis to mediate intratumoral penetration.

Contradicting the hypothesis that a fibrotic ECM is a sufficient physical barrier to prevent T cell infiltration are desmoplastic melanomas. Desmoplastic melanomas are densely fibrotic but also exceptional responders to immunotherapy, with 70% objective response rates and 32% complete responses (138). While these tumors demonstrate a significantly higher mutational burden than PDAC, and pre-existing adaptive immune responses that correlate with PD-L1 expression, this data clearly indicates that T cells are capable of infiltrating a dense, desmoplastic fibrous stroma and that its presence is not sufficient for T cell exclusion. Furthermore, even without engineered protease expression, adoptively transferred T cells can surmount physical barriers to treat experimental PDAC models (139). While ECM composition and rigidity differs between tissue and tumor types, the specific rate-limiting factors for T cell infiltration into fibrotic tissue remain to be determined.

In addition to the effects of desmoplasia on T cell exclusion, angiogenic growth factors such as vascular endothelial growth factor A (VEGF-A), angiopoietin, basic fibroblast growth factor (bFGF), and endothelin-1 (140, 141) attenuate inflammatory-mediated endothelial activation and thus intratumoral vessels exhibit reduced expression of adhesion molecules that would mediate lymphocyte extravasation, such as ICAM, VCAM, and E-selectin (142, 143). For example, bFGF inhibits TNFα/IL-1α-mediated expression of ICAM, VCAM, and E-selectin *in vitro* (144), and VEGF-A disrupts their clustering, therefore decreasing T cell adhesion to ECs (13). Endothelin signaling on ECs increases NO production and subsequent downregulation of adhesion molecules, thus blockade of the receptor increases T cell adhesion and infiltration into tumors (142). Angiogenic signaling from tumor cells also induces FasL expression on tumor associated ECs that limits tumor infiltrating CD8^+^ T cells, presumably through direct killing as demonstrated *in vitro* (145, 146). Consequently, factors that drive the angiogenic switch in tumors simultaneously establishes immunological barriers to limit immune surveillance and facilitates immune escape.

While anti-angiogenic therapy focused on destruction of tumor-associated vessels largely failed in most solid tumor types, adaptation of these strategies utilizing lower, normalizing doses to restore perfusion and adhesion molecule expression has proved more productive (147). Dual angiopoietin and VEGF-A blockade leads to increased T cell accumulation and function in several tumor models and synergizes with anti-PD-1 therapy (120). Furthermore, combination of anti-VEGFR2 and anti-PD-L1 antibodies induced lymphotoxin-dependent emergence of high endothelial venule-like vessels (148), which were necessary for response and are associated with better overall outcome in patients (149, 150). Interestingly, in mouse models, responders to immune checkpoint blockade exhibited rapid reperfusion of intratumoral vessels indicating that intratumoral vascular function may be required for T cell effector function and additionally that checkpoint blockade may directly affect endothelial cells (151). Thus, normalizing the angiogenic tumor vasculature may improve local T cell recruitment generating microenvironments primed to be responsive to immunotherapy. Interestingly, poorly adhesive, angiogenic vessels appear to be largely restricted to intratumoral regions, where they exhibit reduced adhesive properties and elevated expression of immune checkpoints (142). While this geographic vessel heterogeneity may limit infiltration directly into the tumor proper, it still allows infiltration into adjacent stroma perhaps contributing to the dense rings of CD8^+^ T cells observed around tumor nests.

Additionally, antitumor effector and memory T cells restricted to peritumoral stroma may be unable to locate target tumor cells due disrupted chemokine signals. High expression of the T cell attracting chemokines CXCL9, CXCL10, CXCL12, and CCL5 correlates positively with CD8^+^ T cell infiltration across several tumor types (152, 153, 154), indicating that if the tumors express the proper chemokines, T cells can get there. Chemokines, however, can be post-translationally modified by proteolytic cleavage, glycosylation, nitration, or deamination which results in dramatically altered activity (127). When CCL2 is nitrated, by reactive nitrogen species in the TME, for example, T cell infiltration into tumors is hindered and rather remain excluded from the tumor mass (155). In addition to the absence of chemoattractants, secretion of chemokines that serve as chemorepellants may protect tumor nests from T cell infiltration. In a mouse model of PDAC, fibroblast activating protein-expressing CAFs produce CXCL12 that coats tumor cells and prevents CXCR4^+^ CD8^+^ T cells from infiltrating tumor nests and controlling the tumor (156). Administration of AMD3100 (CXCR4 inhibitor) increased T cell infiltration into tumor nests, and synergized with anti-PD-L1 therapy to reduce tumor growth (156). Thus, competing chemokine gradients, initiated and maintained by multiple cell types within the tumor microenvironment, determine lymphocyte positioning and subsequent function.

### Immunological deserts

Finally, immunological deserts are defined as those tumor microenvironments completely lacking T cell infiltrates within tumor nests and in adjacent stroma. Low somatic mutational burden and tumor immunogenicity is likely a significant driver of failed T cell responses in these tumors. However, even in the presence of immunogenic epitopes lymphatic transport and poor DC migration may limit anti-tumor T cell priming in lymphoid organs and thus prevent systemic T cell expansion. In fact, tumors induced or implanted in mice lacking dermal lymphatic vessels fail to activate and accumulate anti-tumor T cell responses (157, 158) and lymphatic vessel density correlates with T cell infiltration in colorectal cancer and melanoma patients (159, 160). Conversely, overexpression of lymphangiogenic growth factors enhances intratumoral inflammation and response to various immunotherapies (42, 161), indicating that lymphatic transport plays an important role in both adaptive immune priming and setting up an inflammatory tumor microenvironment. Thus, the non-hematopoietic stroma may dictate the systemic expansion of anti-tumor immunity and thereby restrict the pool of T cells available for tumor recruitment. Still, downstream of T cell priming, analysis of T cells in synchronous metastases reveals heterogeneous distribution of the existing systemic repertoire (162) indicating additional mechanisms of control. Furthermore, even in the absence of *de novo*, tumor-specific T cell priming, recruitment of pre-existing memory populations should lead to intratumoral accumulation of T cells. Thus, additional factors must limit extravasation and tumor residence of bulk T cell populations. Tissue-specific vascular heterogeneity or dysfunction (stromal and intratumoral) may limit T cell infiltration in a lesion-specific manner and thus contribute to immunological deserts in some and not all metastatic lesions.

## Future directions

Non-hematopoietic cells provide context to *in situ* peripheral tissue immune responses and thus may be critical local signals that determine the switch between protective immunity and immune suppression. Dynamic imaging in animal models continues to reveal the spatiotemporal control tissues exert over infiltrating T cell responses in naïve and inflamed tissues (55). Extension of mechanisms elucidated during normal tissue responses to tumors will provide critical insight into the heterogeneity of T cell recruitment and retention in synchronous metastases in patients (162); may provide novel strategies to improve the efficacy of CAR T cell therapy in solid tumors (163); and provide insight into the multiple immune barriers across solid tumor types. Importantly, careful analysis of tissue-specific differences in immune infiltrate (164, 165), if coupled with tissue-specific vasculature and matrix components through multiplexed imaging technologies, may reveal important environmental context to inform dynamics of intratumoral inflammation and thus response to therapy. Similarly, single cell sequencing, while a powerful tool for extracting novel transcriptional states in tumor, hematopoietic, and stromal cells (143, 166, 167) loses structural information that informs interpretation and thus should be coupled with validation and further discovery in matching tissue sections. The added context may not only improve the prognostic value of extracted biomarker signatures, but will also generate hypotheses for rigorous mechanistic testing in experimental models leading to new strategies for immune modulation and tumor control. Importantly, non-hematopoietic stromal interactions provide inherently local mechanisms of immune control that if targeted, may serve to unleash effector T cell responses and thus revive tumor control.

## Author contributions

RSL and AWL researched the literature, created content, and wrote the manuscript.

### Conflict of interest statement

The authors declare that the research was conducted in the absence of any commercial or financial relationships that could be construed as a potential conflict of interest.
